# Gap balanced adjusted mechanical alignment versus measured resection mechanical alignment: a randomised controlled trial

**DOI:** 10.1007/s00402-022-04487-1

**Published:** 2022-06-12

**Authors:** Hugh Waterson, Robert Walker, Petra Koopmans, Rowenna Stroud, Jonathan Phillips, Vipul Mandalia, Keith Eyres, Andrew Toms

**Affiliations:** 1 Exeter Knee Reconstruction Unit, RD+E Hospital, Exeter, UK; 2 Signidat, Roderwolde, The Netherlands

**Keywords:** TKR, Alignment, Measured resection, Gap balancing, Ligament balancing, Mechanical alignment, Individualised alignment, Functional assessment, Knee outcomes

## Abstract

**Introduction:**

Alignment goals in total knee replacement (TKR) is a topical subject. This study compares the short-term functional outcomes and patient reported outcome measures (PROMs) of two philosophies for knee arthroplasty alignment: measured resection (MR) and an individualised alignment philosophy, with the tibia mechanically aligned and an instrumented gap balancer (GB) to align the femur in both flexion and extension.

**Patients and methods:**

94 knees were enrolled in this randomised controlled trial. The surgical protocol used a MR technique for mechanical alignment or a GB technique for individualised alignment. Primary outcome was quadriceps strength. Secondary outcomes included validated functional tests and PROMs as well as patient satisfaction. Outcomes were assessed pre-operatively, at 6 weeks, 3, 6 and 12 months post-operatively.

**Results:**

At 12-month follow-up, there was no significant difference in the change from baseline mean quadriceps peak torque between the two groups (*p* = 0.988). Significant improvement in the change in range of motion (ROM) in the GB group compared to the MR group at 3 months (13° vs 6° *p* = 0.028) but this improvement was not significant at 1 year (20° vs 17° *p* = 0.21). The functional test of balance showed statistically significant improvement at 6 weeks (*p* = 0.03) in the GB group but this difference was not maintained. PROMs favoured the GB group, with the KOOS pain scoring statistically better (*p* ≤ 0.05) at 6 weeks, 3, 6 and 12 months.

**Conclusions:**

Individualised alignment philosophy utilising a GB technique did not demonstrate an improvement in the primary outcome measure quadriceps peak torque. Improvement was seen in the GB group in PROM pain scores that was significant, both statistically and clinically, out to at least 1 year. Gains that were seen in functional assessment with GB, although significant at some time points, were no longer significant at 1 year and no difference was seen in quads strength. Compared to a MR technique, the individualised GB technique appears to confer some improvement in pain, ROM and some functional tests following TKR in the short-term.

## Introduction

Osteoarthritis (OA) of the knee can be a painful and debilitating condition and total knee replacement (TKR) is an effective treatment to alleviate pain and restore function for patients with OA [1]. Alignment and balance of the components in TKR is critical to the kinematics of the knee; often subtle changes in balance and alignment are only perceived by more active patients or whilst doing more functionally demanding tasks. The introduction of computer navigation improved the accuracy of alignment in the coronal and sagittal planes [2] but this has not translated into improvement in functional outcome or satisfaction [3, 4] and failed to address rotational alignment [5]. With manual instrumentation, there are two techniques commonly used to determine the rotational alignment of the components in TKR: gap balancing (GB) and measured resection (MR). Which technique is superior has been the subject of numerous studies [6], however, there is still continued controversy regarding the best method to determine accurate femoral component rotation.

It has been widely accepted the MR does not restore natural knee kinematics and does not necessarily recreate the natural joint line obliquity, joint height [7], femoral rotation or joint laxity. Accepting this compromise, MR TKR can provide relief of pain, a correction of deformity and a stable predictable joint. MR with Mechanical Alignment (MA) has been the predominant technique for TKR over the last 50 years [8] and it uses independent bone cuts and ligament releases to balance the joint, it also decouples the relationship between the femoral and tibial components in flexion and extension, in particular femoral rotation and flexion. With MR MA alignment is prioritised over balance, there is now substantial literature suggesting that balance may be as important, if not more so [6].

GB uses the patient’s soft tissue envelope to dictate the bone cuts and to achieve this in extension and flexion requires specific instrumentation, navigation or robotics. The compromise with GB is a sacrifice of the historical alignment goals [9].

The subject of TKR surgical philosophy and defining alignment goals remains topical [10] and techniques such as kinematic, natural or anatomical alignment have gained traction over the last few years; leaving patients with preoperative constitutional varus in mild postoperative varus has been associated with improved functional and clinical outcomes [11]. Results from studies utilising alternative alignment techniques have been promising but are not yet clearly inferring long term benefit [12–15].

The Gap balancer (GB) used in this trial (Unity Knee™ EquiBalance™) is a variation on the classic GB technique. It was designed to provide intra-operative confirmation of medio-lateral and flexion–extension soft tissue balance, from which the distal and posterior femoral resections are made, without the need to make ligamentous releases. Where this deviates from previous GB methodology is that the GB is designed to accommodate anatomic alignment principles in extension as well as flexion. The EquiBalance™ GB is unique in that in extension it accommodates up to 3° alignment correction, thereby allowing a balanced cut to be made in extension and provides the opportunity to preserve the natural tibial joint obliquity (0 ± 3°). In this study the tibial cut was made in MA and the distal femoral was made in extension followed by flexion, with the use of the GB.

The aim of this study was to compare the GB technique in individualised alignment based off a MA tibial cut with a standard MR in MA and the effect on functional outcome scores and alignment.

## Patients and methods

Patients were recruited from the waiting list of four consultant knee surgeons (ADT, VIM, KSE, JRP) between June2015 and August 2018. The inclusion criteria were: all skeletally mature patients listed for a primary total knee replacement, who were able to consent for the study participation and could comply with the study protocol follow-up.

Patients were excluded if they had one of the following: a coronal deformity greater than 15°, a fixed flexion contracture greater than 20°, a confirmed OA in the contralateral knee that was anticipated to require surgery within 12 months, prospects for a recovery to independent mobility that would have been compromised by a known co-existent, medical problem. Additionally, any patient known to be a substance abuser or had a psychological disorder that could have affected follow-up care or treatment outcomes were excluded.

**Fig. 1:**
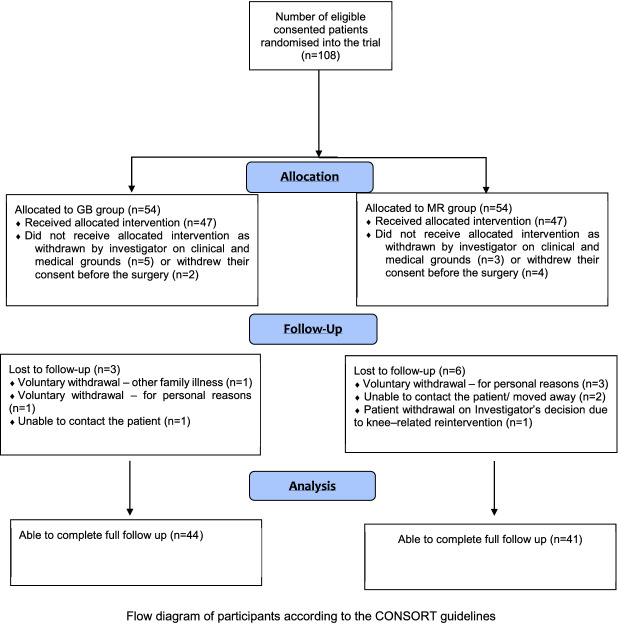
Flow diagram of recruitment and follow up

A flow diagram of the patients in the study, according to the CONSORT guidelines is shown in Fig. 1. A total of 108 patients fulfilled the criteria and were recruited to the study. Of these, 94 (87%) underwent surgery. Fourteen patients withdrew prior to the operation. This was either due their care being transferred to a different facility because of bed pressures, or because they failed their anaesthetic assessment. A total of 9 patients had incomplete follow-up. 44 patients in the GB group and 41 in the MR had complete follow up. Post-operatively one patient in the MR group had a fall and ruptured their patella tendon requiring repair and had to be withdrawn from the functional analysis as they could not complete the tasks.

Randomisation utilised a true random number generator program and sealed envelopes. All patients were reviewed at a pre-operative clinic and at 6 weeks, 3, 6 and 12 months post-operatively by a blinded research physiotherapist.

The primary outcome measure was peak quadriceps torque measured on a digital myometer (MIE Medical Research Ltd, Leeds, United Kingdom). Function was further assessed using the Knee Injury and Osteoarthritis Outcome Score (KOOS) [16], the Oxford knee Score (OKS) [17], and the EuroQol EQ-5D [18], which were collected at each visit. A blinded research physiotherapist assessed the timed get up go (TUG) [19], 6-min walk [20], timed up and down stairs [21], Wii Fit Balance [22], as well as measuring the range of movement (ROM) with a goniometer.

Alignment parameters were measured both pre- and 6 weeks post-operatively on CT and long leg Hip Knee Ankle (HKA) radiographs, that were independently analysed by blinded assessors (HBW and RW) who were not involved in the surgical procedure. As an assessment of reproducibility of these measurements, the interobserver correlations were calculated using Pearson’s r correlation (0.97; *p* < 0.001).

TKRs were performed either with the use of the GB to achieve individualised alignment or with MR MA. A medial parapatellar approach, without the use of a tourniquet, was used and the patella was resurfaced in all cases. The post-operative protocol was identical for both groups. The operations were performed by one of the four consultant knee surgeons who were familiar with both techniques. The trial was registered (NCT02145455) and ethical approval for this study was obtained from the National Research Ethics Service.

**Fig. 2:**
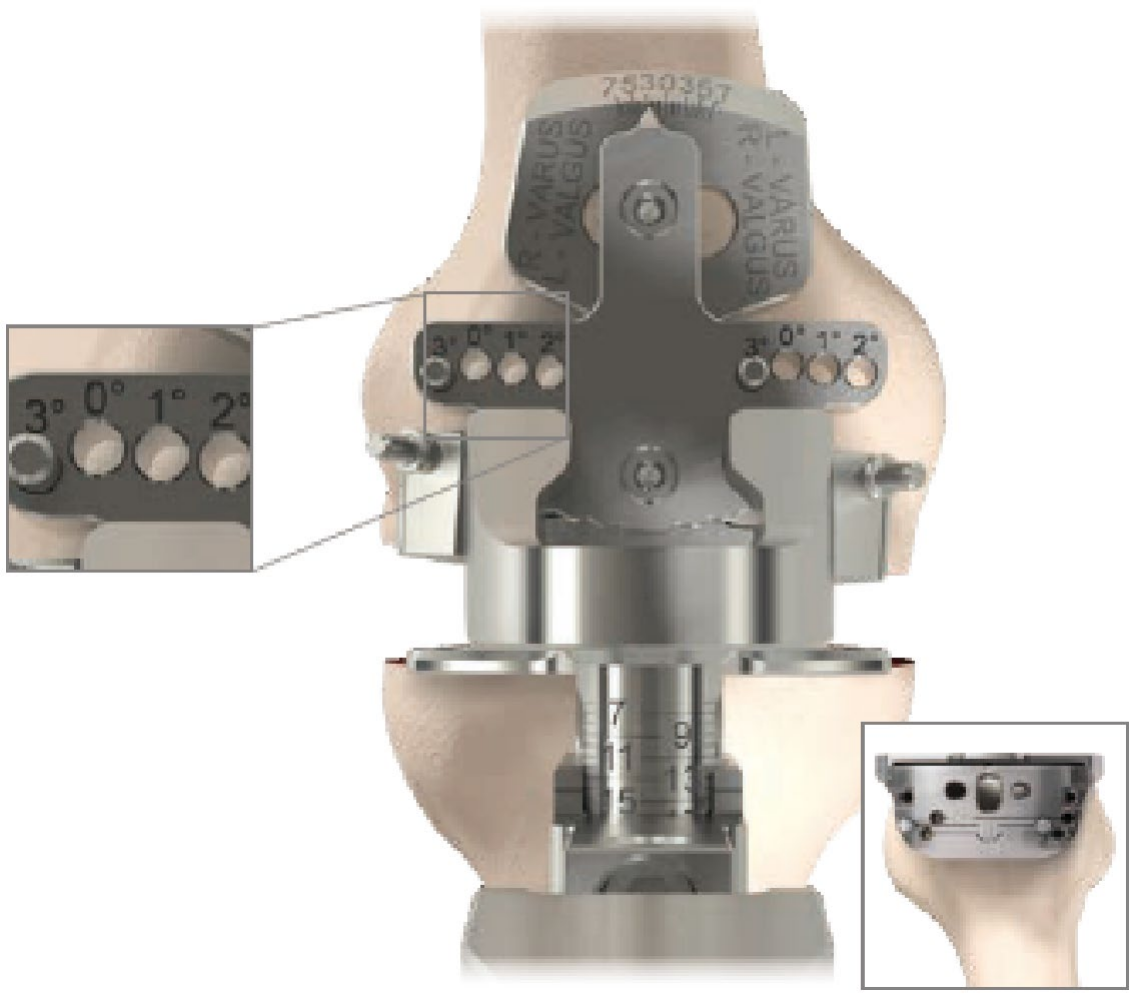
The GB technique (EquiBalance™) illustrating the 3° varus/valgus cut correction that can be made in extension

The implant used in this randomised clinical trial is the Unity Knee™ (Corin Group Ltd, Cirencester, UK) Cruciate Retaining (CR) or Posterior Stabilised (PS) implant and it has instrumentation sets that allow both a MR MA philosophy as well as a GB non-MA philosophy. The Unity knee was introduced in 2013 as part of the Beyond Compliance initiative to promote responsible introduction of new prostheses [23]. The femoral component follows a single radius philosophy and is implanted with cement. The GB technique (EquiBalance™, Corin Group Ltd, Cirencester, UK) uses a manual ligament balancer that allows equal medial–lateral tension in extension through altering the position of the femoral cutting block (Fig. 2).

The GB workflow starts with a tibial cut made in MA using an extra-medullary guide with 3° of posterior slope. A 5 mm distal femoral cut is made off a femoral rod at 5° to allow for stable platform for the ligament balancer instrumentation. The extension balance is assessed and the remainder of the distal femoral cut (making up to 9 mm in total) is made with an adjustment of up to 3° in a varus-valgus plane to achieve a parallel balanced gap. The flexion cut is then made balanced off the tibial cut to achieve medial–lateral balance at the same tension as was achieved in extension. The rotation of the femoral component is not fixed but is dictated by the balance.

The patella was resurfaced using a standard technique to restore the pre-existing patella height. The ability of the extension balancer to adjust the overall leg alignment through an altered distal femoral cut infers that the overall alignment achieved does not aim for a mechanical axis of 0° and can therefore be best termed non-mechanical or individualised.

The MR workflow starts with a tibial cut made in an identical way. The distal femoral cut is made using a 9 mm 5° valgus cut off an intramedullary rod. The femoral flexion cut is made in 3° of external rotation based off the posterior femoral condyles. The patella is resurfaced using the same technique as above. Appropriate soft tissue releases were performed to ensure symmetrical and balanced flexion and extension gaps.

### Statistical analysis

The primary endpoint for the study was a comparison of the power of knee extension via peak torque of quads measured on digital myometer between the two treatment groups. The sample size was powered based on the study by Gomez-Barrena et al. [24] with a difference between the two groups of 8.1 N (SD 13.0), power to 80%, *α* = 0.05 and lost-to-follow up based upon 95% compliance, the sample size was calculated to be 40 in each group.

Functional assessment and patient reported outcome scores are presented as means with 95% confidence intervals (CI)**.** The scores at different visits were compared between treatment groups by means of Analysis of Variance (ANOVA) and for change from baseline by means of Analysis of Covariance (ANCOVA), adjusting for the baseline value, using mixed models. For the ANCOVA we present adjusted means (least square means) with 95% CIs. Differences in alignment between treatment groups were analysed using unpaired t-tests. A *p*-value ≤ 0.05 was defined clinically significant.

## Results

The two groups of patients did not differ with regards to baseline characteristics (Table 1) and baseline values (Table 2). At 1 year, the primary outcome measure of mean quadriceps peak torque (Nm) measured 72.48 (SD 34.57) for GB and 69.13 (SD 23.42) for MR group with no significant difference between the two groups (*p* = 0.61) on ANOVA (Table 2) or ANCOVA (Table 3) testing.

**Table 1:** Baseline characteristics

Variables	Measured resection (*N* = 44) *n* (%)	Gap balancer (*N* = 41) *n* (%)	*p*-value
Age in years			0.670
Number of knees	44	41	
Mean (SD)	70.43 (8.26)	69.77 (6.62)	
Min–Max	50.0–90.0	55.0–85.0	
Median (IQR)	69.0 (66.0–75.0)	70.0 (64.0–74.0)	
Gender			0.837
Female	23 (48.9)	24 (51.1)	
Male	24 (51.1)	23 (48.9)	
Diagnosis			> 0.99
Inflammatory arthritis	1 (2.1)	1 (2.1)	
Osteoarthritis	46 (97.9)	45 (95.7)	
Rheumatoid arthritis		1 (2.1)	
Side of operation			0.679
Left	21 (44.7)	23 (48.9)	
Right	26 (55.3)	24 (51.1)	

Randomised population

**Table 2:** Results of functional tests

	Measured resection	Gap balancer		
Functional test	Mean (95% CI)	Mean (95% CI)	Mean difference (95% CI)	*p*-value
Peak quadriceps torque (N)				
Pre-op	40.79 (33.44–48.13)	50.24 (42.81–57.66)	– 9.45 (– 19.90 to 0.99)	0.075
6 weeks	40.41 (35.34–45.47)	41.90 (36.95–46.85)	– 1.50 (– 8.58 to 5.58)	0.675
3 months	51.42 (44.43–58.40)	57.46 (50.48–64.44)	– 6.04 (– 15.92 to 3.83)	0.227
6 months	60.92 (53.45–68.39)	63.67 (56.38–70.96)	– 2.75 (– 13.19 to 7.69)	0.601
1 year	69.13 (59.79–78.46)	72.48 (63.37–81.59)	– 3.35 (– 16.39 to 9.69)	0.610
Peak hamstring torque (N)				
Pre-op	26.35 (22.09–30.61)	37.54 (33.23 – 41.85)	– 11.19 (– 17.24 to – 5.13)	< .0001
6 weeks	28.13 (24.84–31.42)	29.34 (26.09–32.59)	– 1.21 (– 5.83 to 3.41)	0.605
3 months	34.03 (30.56–37.50)	36.30 (32.83–39.77)	– 2.27 (– 7.18 to 2.64)	0.361
6 months	39.12 (34.56–43.68)	39.41 (34.91–43.92)	– 0.29 (– 6.71 to 6.12)	0.927
1 year	43.59 (39.17–48.01)	44.08 (39.77–48.39)	– 0.49 (– 6.66 to 5.68)	0.875
Wii Fit Balance				
Pre-op	68.89 (63.71–74.08)	67.45 (62.26–72.63)	1.45 (– 5.89 to 8.78)	0.696
6 weeks	65.07 (59.24–70.89)	71.27 (65.51–77.02)	– 6.20 (– 14.39 to 1.99)	0.136
3 months	71.95 (67.38–76.53)	74.20 (69.68–78.72)	– 2.25 (– 8.68 to 4.18)	0.488
6 months	77.41 (74.32–80.50)	76.02 (73.08–78.96)	1.39 (– 2.88 to 5.65)	0.519
1 year	75.13 (71.07–79.18)	74.76 (70.81–78.72)	0.36 (– 5.30 to 6.03)	0.899
Timed up and go (s)				
Pre-op	14.09 (12.03–16.14)	12.55 (10.49–14.61)	1.54 (– 1.37 to 4.45)	0.297
6 weeks	12.09 (10.22–13.96)	12.50 (10.63–14.37)	– 0.41 (– 3.06 to 2.24)	0.759
3 months	10.45 (9.10–11.81)	10.47 (9.13–11.81)	– 0.02 (– 1.92 to 1.89)	0.984
6 months	10.50 (8.43–12.56)	9.94 (7.95–11.93)	0.55 (– 2.31 to 3.42)	0.702
1 year	10.62 (8.99–12.26)	9.16 (7.56–10.75)	1.46 (– 0.82 to 3.75)	0.205
6-min walk test (m)				
Pre-op	238.74 (202.6–274.8)	263.45 (227.3–299.5)	– 24.70 (– 75.73 to 26.32)	0.339
6 weeks	266.24 (228.8–303.6)	298.80 (261.3–336.2)	– 32.56 (– 85.46 to 20.35)	0.225
3 months	334.82 (291.9– 377.6)	360.02 (317.6–402.4)	– 25.20 (– 85.48 to 35.07)	0.408
6 months	367.33 (325.2– 409.4)	378.19 (337.5–418.8)	– 10.85 (– 69.39 to 47.69)	0.713
1 year	358.48 (316.4– 400.4)	389.40 (348.4– 430.4)	– 30.93 (– 89.63 to 27.77)	0.298
Timed stairs (s)				
Pre-op	32.01 (21.12–42.91)	23.12 (12.22–34.01)	8.90 (– 6.51 to 24.30)	0.254
6 weeks	24.45 (20.30–28.60)	24.50 (20.36–28.65)	– 0.05 (– 5.92 to 5.81)	0.986
3 months	17.71 (13.84–21.58)	19.37 (15.55–23.20)	– 1.66 (– 7.10 to 3.78)	0.545
6 months	16.90 (13.77–20.03)	16.58 (13.57–19.60)	0.32 (– 4.03 to 4.66)	0.885
1 year	16.46 (13.75–19.17)	14.10 (11.46–16.75)	2.35 (– 1.44 to 6.14)	0.220

The scores were analysed by means of Analysis of Variance (ANOVA)

**Table 3:** Results of functional tests and KOOS pain score

	Measured resection	Gap balancer			
Functional test	Adjusted mean (95% CI)	Adjusted mean (95% CI)	Adjusted mean difference (95% CI)		*p*-value
Peak quadriceps torque (N)					
6 weeks	− 2.75 (− 23.47 to 17.97)	− 6.41 (− 24.80 to 11.98)	3.66 (− 34.99 to 42.31)		0.783
3 months	11.40 (− 12.24 to 35.05)	7.56 (− 14.41 to 29.54)	3.84 (− 41.24 to 48.92)		0.804
6 months	12.82 (− 36.25 to 61.89)	20.20 (− 24.02 to 64.42)	− 7.38 (− 99.47 to 84.71)		0.763
1 year	25.20 (− 12.92 to 63.33)	25.56 (− 7.18 to 58.30)	-0.36 (− 70.34 to 69.62)		0.988
Peak hamstring torque (N)					
6 weeks	− 4.29 (− 23.75 to 15.18)	− 2.24 (− 21.24 to 16.77)	− 2.05 (− 40.07 to 35.97)		0.838
3 months	1.80 (− 24.18 to 27.78)	4.30 (-22.32 to 30.92)	− 2.50 (− 54.47 to 49.47)		0.888
6 months	1.21 (− 12.21 to 14.63)	12.37 (-1.05 to 25.79)	− 11.16 (− 37.67 to 15.35)		0.273
1 year	7.38 (− 5.05 to 19.81)	16.41 (4.60 to 28.23)	− 9.03 (− 32.97 to 14.91)		0.316
Wii fit balance					
6 weeks	-1.96 (-8.76—4.84)	8.98 (2.04 to 15.93)	− 10.95 (− 20.80 to 1.09)		**0.030**
3 months	7.44 (1.44—13.44)	15.07 (9.03 to 21.11)	− 7.63 (− 16.28 to 1.02)		0.083
6 months	12.45 (8.93–15.97)	14.00 (10.56 to 17.45)	− 1.55 (− 6.60 to 3.50)		0.540
1 year	13.53 (8.41—18.65)	10.72 (5.43 to 16.01)	2.81 (− 4.79 to 10.41)		0.461
Timed up and go (s)					
6 weeks	− 0.65 (− 1.63—0.34)	− 1.05 (− 2.03—− 0.06)	0.40 (− 1.53 to 2.33)		0.617
3 months	0.80 (− 2.24—3.85)	− 6.21 (− 9.18 − 3.24)	7.01 (1.06 to 12.96)		0.031
6 months	− 3.39 (− 6.97—0.19)	− 2.24 (− 5.56—1.08)	− 1.15 (− 7.96 to 5.66)		0.663
1 year	− 3.55 (− 10.46 to 3.35)	− 3.09 (− 9.50 to 3.31)	− 0.46 (− 13.61 to 12.69)		0.918
6-min walk test (m)					
6 weeks	33.75 (− 8.29 to 75.79)	52.35 (13.05–91.65)	− 18.60 (− 82.22 to 45.01)		0.558
3 months	96.51 (53.96 to 139.5)	124.22 (85.10–163.34)	− 27.72 (− 92.90 to 37.47)		0.395
6 months	122.11 (77.1–167.09)	147.45 (105.30–189.61)	− 25.34 (− 94.68 to 44.00)		0.463
1 year	97.10 (53.73–140.48)	155.09 (115.22–194.96	− 57.98 (− 123.8 to 7.88)		0.083
Timed stairs (s)					
6 weeks	5.76 (− 53.64 to 65.15)	− 13.21 (− 73.95 to 47.52)	18.97	(− 99.83 to 137.77)	0.292
3 months	1.37 (− 90.98 to 93.72)	− 20.97 (− 113.3 to 71.38)	22.34	(− 160.2 to 204.93)	0.364
6 months	− 11.55 (− 45.73 to 22.62)	− 10.72 (− 43.29 to 21.85)	− 0.83	(− 66.78 to 65.12)	0.899
1 year	15.78 (− 26.65 to − 4.91)	− 9.56 (− 20.17 to 1.05)	− 6.22	(− 27.44 to 15.00)	0.167
Change in Range of motion (°) from pre-op					
6 weeks	0.07 (− 6.16–6.30)	4.62 (− 1.79–11.03)	− 4.55 (− 11.47 to 2.38)		0.195
3 months	6.39 (1.18–11.60)	12.97 (7.62–18.31)	− 6.57 (− 12.42 to 0.73)		**0.028**
6 months	13.58 (9.53–17.62)	17.93 (13.88–21.97)	− 4.35 (− 8.92 to 0.22)		0.062
1 year	16.78 (12.99–20.56)	19.51 (15.67–23.35)	− 2.73 (− 7.03 to 1.56)		0.209
KOOS pain					
6 weeks	16.03 (8.96–23.09)	25.37 (18.67–32.07)	− 9.34 (− 18.25 to − 0.44)		**0.040**
3 months	26.68 (20.24–33.11)	36.13 (29.91–42.35)	− 9.45 (− 17.77 to − 1.13)		**0.027**
6 months	34.05 (27.74–40.36)	42.51 (36.23–48.78)	− 8.45 (− 16.88 to − 0.03)		**0.049**
1 year	36.11 (30.17–42.06)	46.66 (40.95–52.36)	− 10.54 (− 18.18 to − 2.90)		**0.008**

Bold values denote significant difference ie *p* < 0.05

The scores were analysed by means of Analysis of CoVariance (ANCOVA), change from baseline

**Table 4:** Patient reported outcome measures

	Measured resection	Gap balancer		
Functional test	Mean (95% CI)	Mean (95% CI)	Mean difference (95% CI)	*p*-value
OKS				
Pre-op	19.72 (17.74–21.70)	21.32 (19.34–23.30)	1.60 (− 1.21 to 4.40)	0.261
6 weeks	27.51 (25.10–29.92)	29.8 (27.39–32.21)	2.29 (− 1.12 to 5.70)	0.186
3 months	34.35 (31.98–36.72)	37.32 (34.97–39.66)	2.97 (− 0.37 to 6.31)	0.080
6 months	37.77 (35.27–40.28)	39.88 (37.47–42.30)	2.11 (− 1.37 to 5.59)	0.231
1 year	39.24 (36.83–41.65)	41.14 (38.81–43.46)	1.89 (− 1.46 to 5.24)	0.265
EQ-5D				
Pre-op	68.50 (63.75–73.25)	71.30 (66.54–76.05)	− 2.80 (− 9.52 to 3.93)	0.411
6 weeks	72.07 (67.15–76.99)	73.94 (69.03–78.86)	− 1.88 (− 8.83 to 5.08)	0.593
3 months	80.23 (75.69–84.78)	77.00 (72.51–81.49)	3.23 (− 3.15 to 9.62)	0.317
6 months	79.58 (75.35–83.80)	80.63 (76.55–84.71)	− 1.05 (− 6.93 to 4.82)	0.722
1 year	79.24 (74.60–83.89)	84.37 (79.84–88.91)	− 5.13 (− 11.62 to 1.36)	0.120
KOOS pain				
Pre-op	41.38 (37.55–45.21)	42.83 (39.00–46.66)	− 1.45 (− 6.86 to 3.97)	0.597
6 weeks	59.60 (54.08–65.12)	67.76 (62.24–73.27)	− 8.16 (− 15.96 to 0.36)	**0.041**
3 months	70.53 (65.50–75.57)	79.41 (74.43–84.38)	− 8.87 (− 15.95 to 1.80)	**0.015**
6 months	79.63 (74.55–84.70)	86.93 (82.03–91.83)	− 7.31 (− 14.36 to − 0.25)	**0.043**
1 year	83.71 (78.57–88.84)	90.07 (85.11–95.02)	− 6.36 (− 13.49 to 0.77	0.080

Bold values denote significant difference ie *p* < 0.05

The scores were analysed by means of Analysis of Variance (ANOVA)

There was a significant improvement in the change in ROM seen at 3 months in the GB group in comparison to the MR group (12.97° vs 6.39° *p* = 0.028) (Table 3.). The functional test of Wii Fit Balance showed statistically significant improvement at 6 weeks (*p* = 0.03) in the GB group in comparison to the MR group (Table 3). These differences were no longer statistically significant at longer follow-up.

There was no significant difference at any of the time points between patient groups when assessing their quadriceps and hamstring torque, 6-min walk test, timed stair test EQ-5D and OKS.

The pain component of the KOOS score demonstrated a significantly greater improvement for the GB group, compared to the MR group, at all time-points post-operatively, reaching what is considered the Minimally Clinically Important Change of 8–10 points [25] (Tables 3 and 4). At one year the mean difference in KOOS pain between the two groups was 10.54 (CI − 18.18 to 2.90 *p* = 0.008). There was no significant difference in the other subsections of the KOOS score.

**Fig. 3:**
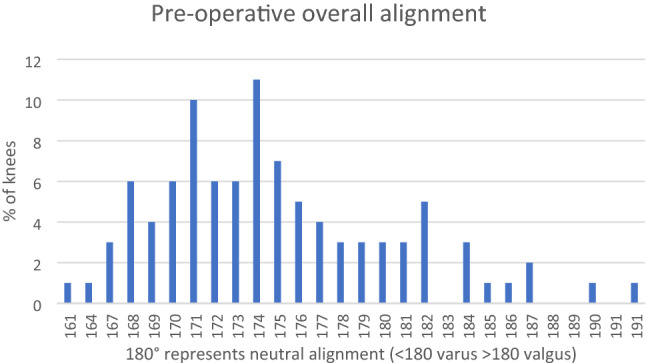
Pre-operative alignment distribution

**Fig. 4:**
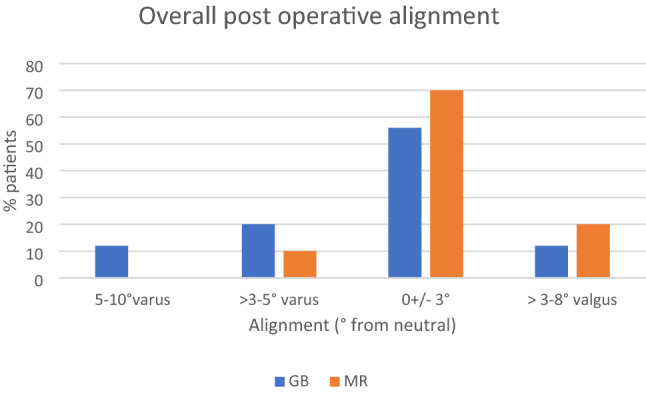
Post-operative alignment in the GB and MR groups

**Fig. 5:**
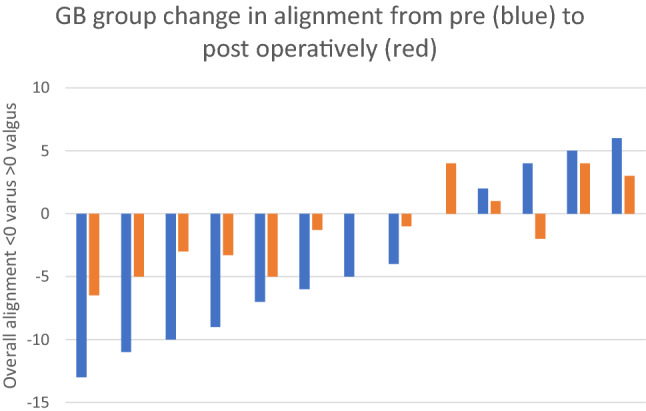
GB group change in alignment from pre- (blue) to post-operatively (red)

**Fig. 6:**
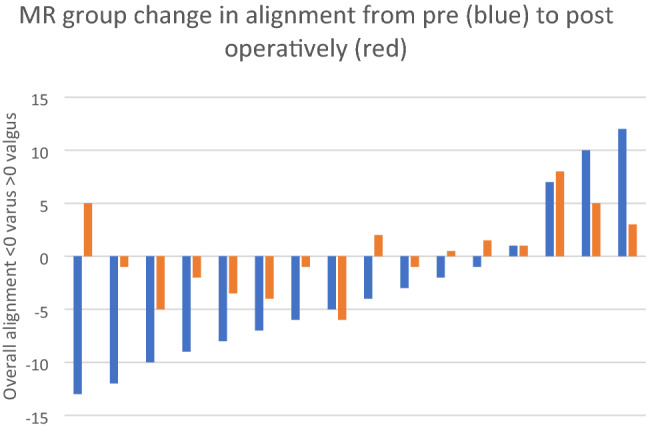
MR group change in alignment from pre- (blue) to post-operatively (red)

Pre-operative assessment of alignment demonstrated an overall alignment mean of 5.2 (SD 5.82) degrees varus with no significant difference in overall alignment between the two groups (*p* = 0.77). The distribution of pre-operative alignment is shown in Fig. 3. Post-operative alignment in the MR group demonstrated an average overall HKA angle of 0.3(SD 3.4) degrees valgus compared to 2 (SD 3.6) degrees varus in the GB group, this reached significance (*p* = 0.05) (Fig. 4). The mean post-operative Medial Proximal Tibial Angle (MPTA) was 90.1 (SD 1.8) degrees in the MR group and 89.6 (SD1.8) degrees in the GB group (*p* = 0.44). The post-operative Lateral Distal Femoral Angle (LDFA) was 89.8 (SD 3.1) degrees in the MR group and of 91.0 (SD 2.9) degrees in the GB group (*p* = 0.22). Figures 5 and 6 illustrate the pre and post-operative change in alignment in the GB and MR groups, and demonstrate less of an overall alignment change in the GB group.

## Discussion

Individualised alignment philosophy utilising a GB technique did not demonstrate an improvement in the primary outcome measure of quadriceps peak torque, however, we found some improvement in early outcomes favouring the GB technique in certain other parameters. Notably the KOOS pain score, was better at 6 weeks, 3, 6 and 12 months in the GB group, although there was no difference in other KOOS subsections. No significant difference at any of the time points was found between patient groups in the 6-min walk test, timed stair test EQ-5D and OKS. ROM and balance were also improved in the GB group post-operatively but this was not maintained at 1 year. The loss of significant difference at 1 year in functional outcomes is similar to other knee trials that have looked at alternative alignment techniques [12].

Our findings suggest that implementation of an individualised alignment strategy using a gap balancing technique may infer some benefit in early functional recovery in patients undergoing TKR. The improvement in KOOS pain score has to be taken in the context of a number of the other functional and PROMs not demonstrating a difference.

Meta-analysis has previously demonstrated that GB may provide better radiographic and clinical outcomes then MR [6], hypothesizing that GB may reflect better anatomical sitting of the components. Previous studies have used GB techniques in flexion, one of the key functions and point of difference of the gap balancer in this trial is that it enables the surgeon to balance the knee in extension by adjusting the angle of the distal femoral cut. The objective intraoperative feedback on alignment enables on-table adjustments to bone resection and implant positioning to achieve balanced and equal flexion and extension gaps without having to perform extensive soft-tissue releases. Direct comparison with previous studies is difficult because of the difference in GB technique and variety of outcome scores that have historically been used. Commonly the Knee Society Score (KSS) and the Knee Society Function Scores have previously been utilized [6], but these are surgeon subjective and have now largely been superseded.

The outcome measures in this study were extensive and incorporated a variety of objective functional outcomes, along with generic and joint specific PROMs. Using a variety of scores has the advantage of being more sensitive in detecting subtle differences in outcome, but could also lead to type 1 errors. The primary outcome in this study (peak quadriceps torque) did not demonstrate any difference between the two groups. We can only hypothesize why specifically the KOOS pain score was significantly improved. No additional soft tissue releases for knee balancing were performed in the GB group in this study. This is in contrast to the MR group where appropriate soft tissue releases were performed to ensure symmetrical and balanced flexion and extension gaps. In the varus knees this included a greater medial release to the posteromedial corner if necessary. In valgus knees this included a posterolateral capsule release and pie crusting of the iliotibial band if necessary. Reduced soft-tissue trauma may have helped reduce post-operative pain as experienced in the GB group.

National Joint Registry of England and Wales data has previously shown that persistent pain following TKR is the strongest predictor of patient dissatisfaction and reduced functional outcomes including the Oxford Knee Score [26] and that pain is the most important prognostic indicator for long-term dissatisfaction following TKA [27]. Interesting, although the KOOS pain score was significant improved in the GB group, this was not reflected in the overall OKS. This likely reflects that the KOOS pain score is more specific to pain with 9 dedicated pain questions, whereas with the OKS, only 5 of the 12 questions are specifically related to pain. Reduced pain scores have been reported in papers using robotic surgery when the technique similarly preserves the soft tissue envelope [28].

The post-operative alignment analysis would support the assertion that the soft tissue envelope was preserved in the GB group. In both groups of patients, the philosophy of the tibial cut was to achieve alignment perpendicular to the mechanical axis of the tibia for insertion of the tibial component. The perpendicular tibial cut in the GB group was to address concerns that a varus tibial resection may predispose to tibial loosening and early failure [29]. There was no significant difference in the LDFA between the two groups although the mean LDFA was slightly more varus (91.0°) in the GB group in comparison to the MR group (89.8°). This was as expected, as the extension gap with the GB technique was not aiming for natural joint line obliquity. The mean pre-operative alignment for both groups was moderate varus (5.2°) and this would be in keeping with a standard knee constitutional varus [30] population, increased by medial OA. The results would suggest that with the GB the constitutional varus alignment has been preserved by maintaining the soft tissue envelope but not at the expense of placing obliquity on the implant. This has the advantages of individualised alignment without the potential risks [29] of the true natural or kinematic alignment philosophy associated with placing the tibial in varus in a limb that is overall in varus.

It should be noted that the outcomes in both groups of patients (OKS > 39 Table 4), are extremely good and compare favourably to other studies [31], this is likely to accentuate any ceiling effect. Looking closely at the results in Table 3, it was the change from baseline results that yielded significant differences in the Wii fit balance at 6 weeks, and it could be hypothesized that proprioception was improved by maintaining a more natural alignment in the GB group helping with balance in the early post-operative period, but as time progressed the patients in the MR group became more accustomed to their new alignment and so relatively improved their balance. The improvement in ROM was only significant in the GB at 3 months, but close analysis of Table 3 demonstrates that at all time points ROM was better in the GB group but not statistically significant.

There are limitations of this study that need to be considered when interpreting the findings. The number of patients in each group was adequate for the power calculation but may have resulted in a type II error. The follow up assessment was time consuming and onerous for the patients and may have led to some loss to follow up. The reported functional outcome measures were not correlated to long-term clinical outcomes beyond 1 year or implant survivorship. By using a large number of tests and a *p* value of *p* < 0.05 to denote significance, this may have led to type 1 error for some of the results. However, it should be noted that even if a *p* value of *p* < 0.01 was used there would still have been a significant difference in KOOS pain score at 12 months. A Bonferroni correction was not performed but if the *p*-value was multiplied with the number of tests performed then if would be difficult to demonstrate any significance.

Individualised alignment philosophy utilising a GB technique did not demonstrate an improvement in the primary outcome measure quadriceps peak torque. Improvement was seen in the GB group in PROM pain scores that was significant, both statistically and clinically, out to at least 1 year. Gains that were seen in functional assessment with GB, although significant at some time points, were no longer significant at 1 year and no difference was seen in quads strength. Compared to a MR technique, the individualised GB technique appears to confer some improvement in pain, ROM and some functional tests following TKR in the short-term.
